# Can Artificial Intelligence Enhance European Emerging Adults’ Psychological Adjustment? A Scoping Review

**DOI:** 10.3390/bs15111483

**Published:** 2025-10-31

**Authors:** Carolina Lunetti, Ainzara Favini, Eugenio Trotta

**Affiliations:** 1 Department of Human Sciences, Guglielmo Marconi University, 00193 Rome, Italy; a.favini@unimarconi.it; 2 Department of Humanities, University of Foggia, 71121 Foggia, Italy; eugenio.trotta@unifg.it

**Keywords:** artificial intelligence, emerging adults, European countries, psychological adjustment, scoping review

## Abstract

Empirical studies support the difficulties European youths encounter when transitioning into adulthood, as well as several economic and social constraints that make the acquisition of a full adult role complex and challenging, with relevant implications for psychological adjustment. In this direction, international research showed the effectiveness of Artificial Intelligence (AI) in promoting mental health adjustment, although most studies are limited to the field of clinical psychology for diagnosing and preventing the onset of pathological problems rather than on non-clinical populations. Furthermore, only a limited number of studies have been conducted in European Countries in comparison to Asian and American countries. Accordingly, this scoping review aims to provide an overview of studies conducted in Europe on AI applications for psychological support to promote psychological adjustment in emerging adults who face the challenges of reaching adulthood, often associated with stress and pressures that increase the likelihood of developing psychological problems. Out of 167 initially selected articles for the period between 2015 and 2025, only six articles were included for the final synthesis, according to explicit inclusion and exclusion criteria, and among them, only three studies were conducted on emerging European adults using different AI tools to provide support to their psychological adjustment. Results from these studies support, first, that despite the significant increase in the AI applications for mental health, their use is still scarce in the European context and specifically to promote emerging adults’ adjustment; second, that despite the scarce applications of AI tools in this sense, results from the few studies are promising regarding the potential AI applications. Future research should better investigate the effects of AI tools to understand their benefits in promoting the mental health of European youths, considering the challenges that they face in going through adulthood.

## 1. Introduction

“Emerging adulthood” is a relatively recent developmental stage, situated between adolescence and young adulthood (18–29 years of age), characterized by individuals experiencing both youth-like dependency and adult autonomy (Arnett, 2000, 2023). This stage is particularly complex and challenging, often associated with pressures and stress, which can negatively affect psychological adjustment. This aspect is compelling in European countries, where the transition to adulthood appears to be even more challenging due to adverse social and economic factors (Žukauskienė, 2015; Isoniemi, 2017). The European situation is worse in comparison to other countries, considering the increasing percentage of people affected by psychological disorders, which has dramatically increased after the COVID-19 pandemic (Li, 2019). In this framework, it highlights the necessity of defining prevention and promotion interventions that, relying on new technologies and tools, aim to enhance positive mental health and adjustment among emerging adults in Europe. Within this broader context, many recent studies support the effectiveness of AI tools and applications in preventing, diagnosing, and treating various psychological disorders. However, most of these studies have been conducted in American and Asian countries, with only partial representation in Europe. Accordingly, the overall aim of this scoping review is to define the state of the art in European AI applications for promoting the mental health of non-clinical emerging adults, thereby better understanding the current European situation and providing important directions for future research. In this framework, we decided to exclude clinical samples from the analyses because specific needs and intervention trajectories characterize these populations. Accordingly, this scoping review’s objectives consist of (1) examining the type of AI tools used to support emerging European adults’ psychological adjustment; (2) examining the methodologies implemented in the selected studies; and (3) examining the primary outcomes, strengths, and limitations of the selected studies. Finally, our focus is on emerging European adults because they face distinctive social and economic barriers in achieving adult roles, leading to a higher vulnerability to psychological distress. In fact, despite increasing AI applications in other continents, evidence from Europe remains scarce, highlighting a significant research gap.

## 2. The Transition to Adulthood in Europe

The transition to adulthood is a specific developmental stage named “emerging adulthood”, which is defined as the “early part of the life course when one leaves behind and gradually adopts a series of adult roles” (Gauthier, 2007, p. 218). Traditionally, the acquisition of the “adult status” was marked by significant changes, such as marriage, parenthood, or securing a stable job. In this framework, it is essential to consider that different countries use different age thresholds to define the challenges of the transition to adulthood (such as the employment age, the age of majority, and the age of school-leaving; Isoniemi, 2017). Therefore, it is not possible to define a specific age range at which an individual can be considered an adult. As stated by Isoniemi (2017), many European countries seem to face this transition as more challenging due to social and economic factors that delay adulthood. For example, in many Eastern and Southern European countries, young adults often still live with their parents. Childbearing also occurs at a very late age, due to economic crises and a lack of opportunities to secure a stable and permanent job. Parental financial support is also necessary to cover the increased costs associated with the required higher education. Empirical studies showed how these changes can cause much psychological distress in emerging adults related to a maladaptive internal response to stress. During emerging adulthood, people face multiple challenges, high demands, and stressors related to changes in work, education, relationships, and place of residence that can undermine both mental and physical health (Matud et al., 2020; Villar et al., 2022). For these reasons, it is of fundamental importance to better investigate the potential interventions that can promote emerging European adults’ mental health and effectively act to prevent psychological problems.

## 3. AI to Promote Mental Health

### 3.1. The AI Proliferation to Prevent Psychological Problems

AI is defined as a set of computer/machine techniques that utilize artificial agents capable of perceiving their surrounding environments and performing actions to maximize the likelihood of achieving specific goals (Venkatesh, 2022; Wang, 2019). Within the realm of AI tools, machine learning has garnered significant attention, particularly in its three major types of tools (Mohr et al., 2017): supervised/semi-supervised machine learning, unsupervised machine learning, and deep learning. AI methods can process vast amounts of data in real-time, thanks to advances in computational power and big data analytics (Venkatesh, 2022). This has led to the widespread implementation of AI-based systems in our everyday lives, from facial recognition systems to voice assistant tools (Su et al., 2020; Venkatesh, 2022).

One use that has been increasing significantly in recent years is the application of AI to the prevention, diagnosis, and treatment of various psychological problems (Marx et al., 2017). Regarding mental health promotion, human–artificial intelligence interaction provides important insights and applications in various fields, including advanced diagnostic approaches, personalized therapies based on objective indicators, and therapeutic platforms tailored to the specific needs of users (Olawade et al., 2024). Among the most relevant applications, these technologies can also analyze a user’s language or text, examine micro-facial expressions of emotions as indicators of mental health status, and analyze databases of electronic medical records to create predictive models for personalized treatments and mental health promotion (Johnson et al., 2021). For example, natural language analysis techniques allow for the extraction of relevant information from both written and spoken language, as indicators of emotional state (Zhang et al., 2022). In this direction, artificial intelligence has also exploited the analysis of chats and activity logs in social media to examine changes in emotional state. Additionally, voice analysis can detect alterations and predict potential psychological issues (Flanagan et al., 2021; Tracy et al., 2009).

### 3.2. The European Context

Mental health problems are currently one of the most significant issues in the European context. According to the WHO (World Health Organization), in 2021, more than 150 million people from Europe suffered from at least one mental health problem, and of these, only one in three received adequate treatment. To improve this critical situation and promote mental health, several actions and programs have been undertaken at the European level, including the European Program of Work 2020–2025, promoted by the WHO. However, it is important to emphasize that, despite the actions undertaken, these programs often fail to utilize important tools, such as AI applications. In fact, since around 2010, artificial intelligence (AI) has been increasingly utilized to develop methods and tools that enhance the diagnosis, treatment, and prevention of mental illnesses, underscoring the need for further research and development. However, most applications have been concerned with Asian or American countries and, only in a significantly smaller number of cases, in European countries (Li, 2019). Considering AI applications to mental health in Europe, studies also report several limitations, such as insufficient sample sizes and a limited selection of clinical participants (Tornero-Costa et al., 2023). These limitations underscore the need to understand the current state of the art in AI applications for mental health in contexts where they are not yet widely applied, specifically in European countries, to better understand how to overcome current limitations and identify future opportunities and prospects.

### 3.3. The Present Study

As previously reported, over the last decade, the widespread use of new internet technologies has facilitated the development of various internet-based interventions aimed at promoting psychological well-being and reducing psychological problems (for a review, see Sîrbu & David, 2024). However, most previous studies utilized mobile web apps that already existed, and only a minimal number of interventions and research considered AI tools (e.g., Li Pira & Ruini, 2025; Walsh et al., 2024).

Of the relatively new AI instruments developed for mental well-being and adjustment, a variety of studies focused on specific conditions, such as preventing psychological distress and maladjustment in the general population during wars (e.g., Lahutina et al., 2024), preventive or intervention strategies targeted specific psychopathological conditions (e.g., Horwitz et al., 2024), or for adolescent populations (e.g., Viduani et al., 2023). Most of the existing studies that adopted various AI tools focused on the possibility of detecting psychopathological conditions by using deep learning, machine learning networks, or virtual agent–human interactions (Mohr et al., 2017; Su et al., 2020). To our knowledge, minimal research has considered emerging adults as target populations, and most existing studies have been conducted in the United States and Asia, rather than in Europe.

Accordingly, the overall aim of the present scoping review was to give an overview of the available studies that adopt AI instruments as a vehicle to foster psychological well-being and mental health in emerging European adults. In addition, considering the fact that EU countries share a common regulatory and political ground, within which there are disparities in terms of socio-economic resources, we also aim to investigate if the AI tools are contextualized to the countries’ social characteristics.

## 4. Materials and Methods

### 4.1. Search Strategy and Literature Search

Our systematic literature search was conducted in March 2025, following the framework by Arksey and O’Malley (2005), using three databases: PubMed, PsycINFO (via EBSCOhost), and Scopus. We limited the identification to the topic section (title, abstract, keywords), using a combination of several terms related to psychological well-being, university students, AI tools, and European countries (see the Supplementary Materials for details). Next, we searched further articles, consulting the references of the selected articles (backward analysis) and the studies that cited the same articles (forward analysis). We defined specific advanced queries for each database using different combinations of the Boolean operators “AND” and “OR.” (This study was conducted according to the PRISMA-ScR guidelines for scoping reviews (e.g., Moher et al., 2009; Tricco et al., 2018; Swartz, 2021).

We stored and analyzed, qualitatively and quantitatively, the items found by using the CADIMA online platform for reviews (Kohl et al., 2018). The study was registered on the Open Science Framework (https://osf.io/s9g5a—7 May 2025). Finally, consistently with the aims of a scoping review (Munn et al., 2018), no formal quality appraisal tool (e.g., CASP) was applied, but we only focused on mapping the aims, characteristics, and methodologies of the included studies.

### 4.2. Inclusion and Exclusion Criteria

The inclusion and exclusion criteria for the studies were defined before the literature search. The following criteria were considered:

- Published papers in the last 10 years (i.e., from 2015 to 2025).
- Works written in English and published, excluding preprints, unpublished manuscripts, or manuscripts under review.
- Studies conducted in the European countries (i.e., Austria, Belgium, Bulgaria, Croatia, Cyprus, Czechia, Denmark, Estonia, Finland, France, Germany, Greece, Hungary, Ireland, Italy, Latvia, Lithuania, Luxembourg, Malta, Netherlands, Poland, Portugal, Romania, Slovakia, Slovenia, Spain, and Sweden). We also included studies conducted in the UK published when it was still part of the EU (before January 2020).
- Studies conducted with emerging adult populations, excluding clinical samples.

In terms of qualitative research questions, we defined several theoretical aims, as follows:

1. To examine AI methods for preventing and predicting emotional problems in a European sample of emerging adults.
2. To identify AI and web-based protocols that analyze and integrate psychological and physiological information for examining emerging European adults’ mental health.
3. To examine whether AI methods can foster emotional functioning in emerging European adults by adopting specific therapy techniques within the cognitive and behavioral approach.

### 4.3. Study Selection Process

According to PRISMA guidelines, we screened the 167 identified records by assessing whether each title could be relevant to the scope of our study. Two independent researchers evaluated the title, resulting in a total of 86 records being excluded from further procedures. Then, the two independent researchers evaluated each remaining record by the relevance of the abstract, excluding the other 57 records. Another record was excluded because the full text was not available, so a total of 23 papers passed to the next step of eligibility evaluation. This preliminary screening procedure was conducted in accordance with the inclusion/exclusion criteria established for developing the queries.

In the eligibility step, the full text of each record was carefully evaluated by two independent researchers. The inter-rater agreement for the entire eligibility step was calculated according to previous indications (e.g., Rau & Shih, 2021) as the percentage of agreement, which was 92%. In the final stage of the comprehensive text evaluation, for records that were systematic/narrative reviews and/or meta-analyses, we conducted a preliminary qualitative analysis of the papers included in these previous works, identifying two new records. In evaluating the accountability of each study, we excluded all theoretical papers, commentaries, and papers that did not provide clear information about the sample used (i.e., those with fewer than three records). Moreover, we excluded all the studies that were conducted in countries outside the European context (i.e., two records) and those that did not include any AI tool in their design or methodology (i.e., nine records). Finally, we excluded all studies that did not really consider the population of emerging adults (i.e., three studies).

**Figure 1:**
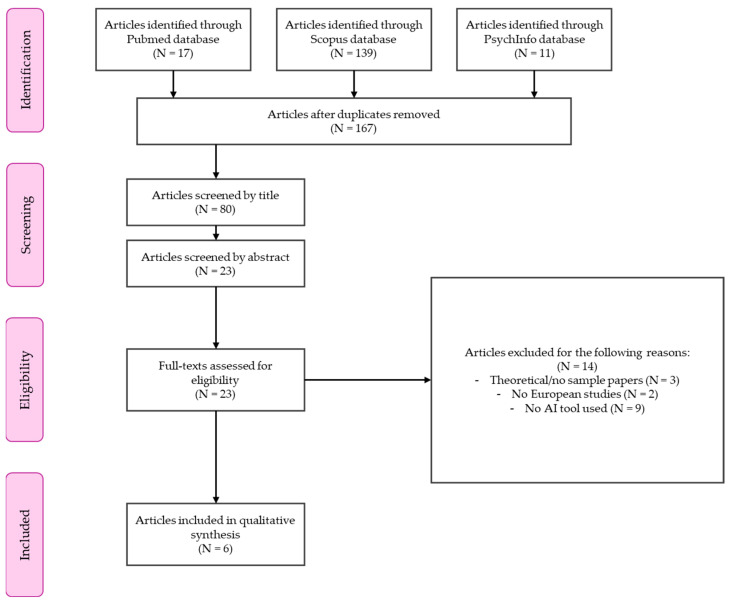
Flowchart of the selection procedure, bibliographic search from January 2015 to March 2025.

The entire selection process is summarized in Figure 1.

### 4.4. Data Extraction

To summarize the findings of the selected records, a researcher extracted the following information for each publication: the year of publication, the type of sample and the available descriptive information, the country/ies in which each sample was collected, the type of AI tool utilized in each study, the principal measures adopted, the design and the methodology of each study, the identified predictors of each work, and the main findings. The entire data extraction procedure was conducted by one researcher and supervised by two other researchers to ensure its validity and reliability.

## 5. Results

We conducted a narrative synthesis of the selected articles, where findings were described thematically rather than pooled statistically, by considering (1) characteristics of the included studies, (2) populations considered, (3) AI tools and methodology, and (4) psychological outcomes related to adjustment. This synthesis is named qualitative synthesis (Popay et al., 2006). A total of six studies were considered for the qualitative synthesis, which is detailed below, dividing the selected records into those meeting each of the main inclusion criteria previously defined.

### 5.1. Characteristics of the Included Studies

Of the six studies considered for qualitative analysis, all were published in the last five years, with publication years ranging from 2017 to 2025. The studies mainly considered one country (i.e., 66.7% of the studies), which were predominantly conducted in the UK (two studies (i.e., Marx et al., 2017; Litchfield et al., 2023), followed by Italy (two studies (i.e., Li Pira & Ruini, 2025; Desideri et al., 2019)), while 33.3% of the studies considered samples derived from more than two countries. They always included the UK as one of the mentioned nations (Koutsouleris et al., 2021; Greyling & Rossouw, 2025). These results are summarized in Table 1.

### 5.2. Populations Considered

As regards the samples involved in the studies considered, a small percentage included clinical populations affected by anxiety and/or depression (i.e., 20% of the samples). Two studies considered a sample of undergraduate students in psychology or other types of programs (e.g., Desideri et al., 2019; Li Pira & Ruini, 2025). Another study considered a sample of emerging adults, specifically staff members of English Care Institutions and organizations (e.g., Litchfield et al., 2023), and one study derived its results from a large dataset provided by Google Trends^TM^ (Greyling & Rossouw, 2025). As regards the age ranges of the samples involved in the studies, a third study did not provide information about the age of participants (e.g., Greyling & Rossouw, 2025; Litchfield et al., 2023; Marx et al., 2017); another study considered emerging adults samples even if composed of clinical participants that were younger than 25–29 years (i.e., Desideri et al., 2019; Koutsouleris et al., 2021; Litchfield et al., 2023; Li Pira & Ruini, 2025). Lastly, regarding participants’ gender, in two cases, information about gender was not available (i.e., Greyling & Rossouw, 2025; Litchfield et al., 2023), and 50% of the studies considered samples in which females were more predominant than males (i.e., Desideri et al., 2019; Koutsouleris et al., 2021; Li Pira & Ruini, 2025). These results are summarized in Table 1.

### 5.3. AI Tools and Methodology

Of the studies considered, half adopted a cross-sectional design (50%), and half employed a longitudinal design (50%). The cross-sectional studies utilized heterogeneous design methods, such as a qualitative design for the assessment of participants’ feelings regarding the adoption of AI tools (e.g., Litchfield et al., 2023), quantitative AI-research methods to investigate comorbidity patterns among psychological diseases (Marx et al., 2017), quantitative AI-research methods to identify an index of psychological well-being (Greyling & Rossouw, 2025), or a cross-sectional experimental design (Desideri et al., 2019). For example, Desideri and colleagues (Desideri et al., 2019) employed a pre–post design to investigate participants’ interactions in two conditions (i.e., human–human and robot–human), testing possible differences in the influence on emotional processes of interacting with a person versus a robot. Marx and colleagues (Marx et al., 2017) adopted a machine learning data-mining approach to investigate the comorbidities of depressive problems with a wide range of psychological and organic diseases. The design methods were also heterogeneous for longitudinal studies, which varied from experimental designs (Li Pira & Ruini, 2025) to clinical longitudinal cross-cultural designs (Koutsouleris et al., 2021). For example, Li Pira and Ruini (2025) adopted a pre–post intervention and follow-up approach to test the effectiveness of the VR intervention in fostering well-being and reducing psychological distress.

In terms of AI tools included in the studies, more (66.7%) utilized various types of machine learning and data-mining techniques, such as Bayesian algorithms (e.g., Marx et al., 2017), multimodal machine learning models (e.g., Koutsouleris et al., 2021), or linear regression machine learning models (Greyling & Rossouw, 2025). The remaining percentage (33.3%) incorporated AI into software, online platforms, mobile apps, or robots (e.g., Desideri et al., 2019; Litchfield et al., 2023). These results are summarized in Table 2.

### 5.4. Psychological Outcomes Related to Adjustment

As mentioned in the previous paragraphs, only three studies considered normative samples of emerging adults. (e.g., Desideri et al., 2019; Li Pira & Ruini, 2025; Litchfield et al., 2023). Of the three papers, the study conducted by Desideri and colleagues aimed to investigate emotion-related cognitive processes (Desideri et al., 2019). They focused on emotional variables, both psychologically (e.g., anxiety, positive and negative affect, motivation, and mood, as well as non-verbal emotional behaviors) and physiologically (e.g., Heart Rate variability), as well as on cognitive processes related to interaction with someone else (e.g., cognitive workload). In terms of main findings, this study revealed that in robot-human interaction, participants were slightly more anxious about the situation than in human–human interaction, which is more related to negative affect. Additionally, in terms of non-verbal behaviors, robot interaction elicited more “time looking” behaviors. In contrast, in human interactions, more avoidant behaviors were elicited, indicating no differences in cognitive processes (Desideri et al., 2019). The second study aimed to address the potential benefits of a VR intervention in reducing psychological distress in a sample of college students. This work also focused on emotional issues (e.g., positive and negative affect, depressive and anxiety problems; Li Pira & Ruini, 2025). After the intervention with the VR program named “H.O.M.E.” (the acronym for How to Observe and Modify Emotion), they found a significant reduction in stress symptoms related to anxiety and depression, as well as an increase in well-being among participants, supporting the effectiveness of the program in reducing emotional distress (Li Pira & Ruini, 2025). The last study qualitatively analyzed individuals’ perceptions of the use of Sensor-based Artificial Intelligence Technology for work purposes (Litchfield et al., 2023). The main findings suggest that, at an individual level, there were still many concerns about the usability of AI tools for managing everyday work tasks, due to the complexity of the instruments and a lack of information on the tools’ functioning (Litchfield et al., 2023).

Of the papers that considered clinical samples, the study by Koutsouleris and colleagues (Koutsouleris et al., 2021) focused on emerging adults in the condition of Clinical High Risk (i.e., CHR) and Recent Onset Depression (i.e., ROD), who are potentially vulnerable to the manifestation of psychosis. Descriptively, they found that the two conditions were significantly associated and that both predispose to a higher incidence of psychosis (Koutsouleris et al., 2021). Adopting machine learning models, they also found that a variety of clinical and neurocognitive variables significantly predicted the emergence of psychosis in the two groups, as issues in facial emotion recognition, lower positive symptoms, motor disturbance, non-supportive family contexts for psychological factors, polygenic risks for the whole genome P for heritability factors, and altered gray matter volumes as sMRI factors, supporting for the stratification of the predictors across different clinical–neurocognitive and genomic factors (Koutsouleris et al., 2021). Marx and colleagues (Marx et al., 2017) adopted a similar approach by identifying comorbidity patterns of depression using machine learning Bayesian mapping models. Across different models, depression showed strong comorbidity patterns with anxiety problems, other depressive problems (e.g., bipolar), strong comorbidity patterns with metabolic disorders, and a variety of neuro-cognitive problems, such as chronic fatigue or migraine (Marx et al., 2017). Lastly, two papers that did not provide information about the characteristics of the samples considered (Greyling & Rossouw, 2025; Litchfield et al., 2023), one study aimed to develop an index of Happiness by investigating big data information (Greyling & Rossouw, 2025). Findings of the first study, which employed machine learning techniques, evidenced that AI can adequately identify a general index of psychological well-being at the population level (Greyling & Rossouw, 2025). These results are summarized in Table 3.

## 6. Discussion

Empirical studies emphasize the difficulties that emerging adults very often face in acquiring the full adult role due to societal and contextual factors that impede their ability to reach milestones that in the past defined the transition to adulthood, such as marriage, parenthood, and obtaining a stable job (Arnett, 2000). Considering the efforts that individuals put into facing these challenges is of fundamental importance in preventing the psychological maladjustment caused by the pressure and stress that emerging adults face. This emergency is even more pronounced in European Countries, where, due to recent economic crises and the increasing percentages of people who suffer from psychological problems, it is more challenging to transition to adulthood (Isoniemi, 2017).

Considering this critical situation, the overall aim of this scoping review was to provide a picture of the available studies conducted in European Countries on AI applications to support emerging adults’ mental health and adjustment.

The specific objective consists of describing the characteristics of these studies in terms of AI tools used, methodologies, main outcomes, strengths, and limitations. To this aim, we review existing research by selecting studies that have been conducted within the last ten years in Europe, written in English, and within the college students’ population, with no age or sample limitations, excluding clinical samples.

Initially, we identified 167 articles and ultimately selected only six. We analyzed the final six studies in terms of sample characteristics, methodology, study design, and AI tools used. All the selected studies were published within the last five years and focused on just one country (primarily in the UK and Italy). Regarding the adopted methodologies, half of the selected studies used a cross-sectional design characterized by heterogeneous methods; the other half of the selected studies were longitudinal and included interventions to help participants in dealing with their psychopathological symptoms. A deeper analysis of the implemented AI tools suggests that half of the studies used various types of machine learning and data-mining techniques, such as Bayesian algorithms, multimodal machine learning models (e.g., Koutsouleris et al., 2021), or linear regression machine learning models (Greyling & Rossouw, 2025). The other half incorporated AI into software, online platforms, mobile apps, or robots (e.g., Desideri et al., 2019; Litchfield et al., 2023).

Finally, only three studies conducted in the last decade directly focused on emerging European adults (Desideri et al., 2019; Li Pira & Ruini, 2025; Litchfield et al., 2023).

The first study was conducted by Desideri and colleagues (Desideri et al., 2019) to examine emotion-related cognitive processes in robot–human interaction, supporting a higher attentional engagement and higher anxiety compared to human–human interaction. The second study was conducted by Li Pira and Ruini (Li Pira & Ruini, 2025), aiming to address the possible positive effect provided by a VR intervention in the reduction of psychological distress in a sample of university students; this study showed a significant reduction in stress symptoms related to anxiety and depression, as well as an increase in well-being among participants, supporting the effectiveness of the program in reducing emotional distress (Li Pira & Ruini, 2025).

The third study examined individuals’ perception of sensor-based artificial intelligence technology in the workplace (Litchfield et al., 2023). The key findings indicate that, on an individual level, concerns persist about the practical use of AI tools for managing daily work tasks. These concerns are primarily attributed to the complexity of the technology and a general lack of understanding about how these tools operate (Litchfield et al., 2023). Considering these findings in light of our specific research questions, this review suggests that, regarding the most used AI tools, there is no consistency among the studies that met our inclusion criteria; also, the methodologies were different, with some studies focused just on the examination of target variables, while other studies include intervention; finally, the considered outcomes were also different because some studies are based on specific emotional and psychological factors, while other studies are based on the process involved in the human machine interaction. Therefore, results from this review indicate a fragmented landscape characterized by inconsistent findings, heterogeneous methodologies, and a limited number of studies conducted on non-clinical European youths. In summary, the limited number of studies on the use of AI to support the psychological well-being of emerging European adults highlights that the transition to adulthood is still not systematically defined in the European context. Despite the increasing interest, the current literature reveals that both traditional and digital interventions for emerging adults remain scarce, primarily focusing on specific contexts (e.g., clinical settings). This scarce evidence confirms that the transition to adulthood varies significantly among people and that AI tools have so far not been widely adapted to the coping mechanisms (coping, resilience, social support) typical of young people at this stage. Overall, this study’s findings show that AI tools hold potential for promoting psychological adjustment among emerging European adults; however, the limited number and heterogeneity of studies indicate an initial interest in this research area. This suggests the need to investigate this field further, also integrating AI into preventive and community-based interventions, which could represent a promising direction for future research.

### Conclusions and Implications

There are some important limitations and implications that this scoping review must acknowledge.

First, the number of studies is very limited: while we initially identified 167 studies, only six met our inclusion criteria, and among these, only three involved nonclinical emerging adults.

Second, the small sample size of the included studies limits the generalizability of the findings. Despite these two important limits, we tried to maintain a rigorous search methodology, including the PRISMA flow chart and checklist, and an adequate and transparent search strategy (Munn et al., 2018; Peters et al., 2015).

Finally, this scoping review provides important directions for future research, suggesting the importance not only of expanding studies in this field to larger and more representative non-clinical samples, but also to develop longitudinal and experimental interventions to promote more involved and personalized approach on the use of AI to respond to the diversified needs of the transition to adulthood and to integrate those into community/educational contexts.

## Figures and Tables

**Table 1:** Results of the studies published from 2015 to 2025 based on the inclusion criteria adopted: Study descriptive statistics and sample information.

Reference *	Country	Sample Characteristics
Desideri et al. (2019)	Italy	Type = Undergraduate bachelor’s psychology students N = 29; 59% Females M_age_ = 24.61; *SD* = 2.42; age-range = 18–35
Greyling and Rossouw (2025)	UK and The Netherlands	Type = Google Trends^TM^ dataset Further information not provided minimum age = 16
Koutsouleris et al. (2021)	Finland, Germany, Italy, Switzerland, UK	Type = Clinical patients and control group N = 668; 53% Females M_age_ = 25.1; *SD* = 5.8; age-range = 15–40
Li Pira and Ruini (2025)	Italy	Type = College students N = 16; 87% Females M_age_ = 23.2; *SD* = 2.9; age-range not provided
Litchfield et al. (2023)	UK	Type = Staff members of care institutions N = 18 Age and gender not provided
Marx et al. (2017)	UK	Type = Clinical patients N = 117′392; 55% Females M_age_ = not provided

* Papers are reported in alphabetical order.

**Table 2:** Results of the studies published from 2015 to 2025, based on the inclusion criteria adopted: methodology and AI tools utilized.

Reference *	Design	AI Tool	Methodology
Desideri et al. (2019)	Experimental study with pre-post measures	AI robot	Independent and paired sample *t*-tests, ANOVA, and correlations to analyze human–robot interactions and compare with human–human interaction in influencing the emotional process
Greyling and Rossouw (2025)	Cross-sectional study	Machine learning	Machine learning algorithms to develop and validate a population-based index of emotional well-being in English and Dutch, integrating large-scale datasets with survey data
Koutsouleris et al. (2021)	A clinical longitudinal cross-cultural study	Machine learning	Multimodal machine learning models, incorporating psychological and physiological data, to predict the emergence of psychosis in psychiatric patients with high-risk diseases and recent onset of depressive problems
Li Pira and Ruini (2025)	An experimental study with pre-post-follow-up measures	Software	Analysis of variance (MANOVA and one-way ANOVA) to analyze the impact of VR treatment on psychological adjustment
Litchfield et al. (2023)	Qualitative study with semi-structured interviews	Sensor-based AI (SAT) system	NASS framework to analyze data derived from semi-structured interviews to evaluate the selection and implementation of the SAT qualitatively
Marx et al. (2017)	Cross-sectional study	Text mining and machine learning	Probabilistic graphical models in the Bayesian framework to construct multimorbidity maps among the psychopathological diseases

* Papers are reported in alphabetical order. Notes: AI = artificial intelligence; MANOVA = multivariate analysis of variance; ANOVA = univariate analysis of variance; VR = virtual reality; SAT = sensor-based artificial intelligence technology; NASS = non-adoption, abandonment, scale-up, spread, and sustainability framework.

**Table 3:** Results of the studies published, from 2015 to 2025, based on the inclusion criteria adopted: Measures and main findings.

Reference *	Measures	Predictors/ Outcomes	Main Findings
Desideri et al. (2019)	- State-Trait Anxiety Inventory (STAI) Visual Analog Scale (VAS) - Positive Affect and Negative Affect Scale (PANAS) - Ethological Coding System for Interviews (ECSI) - Heart Rate variability (HR, Ottaviani et al., 2015) - NASA Task Load Index (TLX) - Wechsler Adult Intelligence Scale (WAIS)	*Predictors:* - Human–human interaction vs. Human-robot interaction *Outcomes*: - State and Trait Anxiety (STAI) - Motivation and mood (VAS) - Positive Affect (PA) and Negative Affect (NA) - Non-verbal behaviors (ECSI) - Heart rate variability (HR) - Cognitive Workload (TLX) - Recall and Subtraction cognitive tasks (WAIS)	*Robot-human condition*: significant correlation between state anxiety and PA; increase in sympathetic HR activation; higher “time looking” non-verbal behaviors. *Human–human condition*: significant correlation between trait anxiety and NA; higher “gaze aversions” non-verbal behaviors.
Greyling and Rossouw (2025)	- 69 emotion-specific words were data-mined according to literature - True Happiness	*Predictors*: - Emotion-specific words - True Happiness *Outcomes*: - Estimated Happiness - Estimated Happiness vs. True Happiness Happiness invariance across time (2021–2022) - Cross-cultural validation of emotion-specific words as predictors of Happiness	26 emotion-specific words were identified, of which the negative emotion words are the best predictors of Happiness. The machine-learning estimated Happiness adequately overlaps with the true measured Happiness. Trends for Happiness consistently reflect trajectories over The cross-cultural validation of the 26 emotion-specific words required integrating country-specific words to adequately predict Happiness in the Netherlands, resulting in 23 words.
Koutsouleris et al. (2021)	- Structured Interview for Psychosis-Risk Syndrome - Schizophrenia Proneness Instrument—Adult (SPI-A) - Childhood Trauma Questionnaire (CTQ) - Trail-Making Test (TMT) - Diagnostic Analysis of non-verbal Accuracy (DANVA) - Semantic and Phonetic Verbal Fluency (S/P-VF) - MRI scanning data - Genotyping DNA data (PRS)	*Predictors*: - Psychosis Risk - Vulnerability to Schizophrenia (SPI-A) - Childhood trauma experiences (CTQ) - Processing speed, cognitive flexibility, sustained attention, and inhibitory control (TMT-B) - Facial emotional recognition (DANVA) - Verbal fluency (S/P-VF) - MRI - DNA Genomes (PRS) *Outcomes*: - Trajectories of psychosis in two conditions (CHR and ROD)	Descriptively, CHR and depression were highly correlated. The complete clinical-neurocognitive model of psychosis prediction was the best machine learning model and attested different trajectories of psychopathology in CHR and ROD cases, as well as in psychosis and no-psychosis cases. The model was confirmed cross-culturally (Germany, Finland, Switzerland, and Italy).
Li Pira and Ruini (2025)	- Depression Anxiety Stress Scale-21 (DASS-21) - Mental Health Continuum (MHC) - Positive Affect and Negative Affect Scale (PANAS) - Qualitative data for software feasibility and acceptability	*Predictors:* - VR treatment *Outcomes*: - Depression/Anxiety Stress (DASS) - Positive Affect (PA) and Negative Affect (NA) - Mental Health Continuum (MHC)	Significant reduction in DASS and increase in well-being (i.e., MHC and PA). NA did not change significantly
Litchfield et al. (2023)	- Semi-structured interview according to NASSS framework	*Outcomes*: - Condition of SAT implementation - Technology of the SAT system - Potential value of the SAT implementation - Adopters of the SAT system - Organization’s approach to SAT implementation - Wider system attitude toward the SAT system	The conditions of SAT implementation were perceived as confusing. The system was evaluated as complicated to use. The staff perceived their roles as complex. Their organizations did not provide sufficient training in using the SAT systems. The wider societal policies required infrastructure that demanded the adoption of more complex technologies.
Marx et al. (2017)	- UK Biobank disease categories with almost 1% prevalence using Mental Health Questionnaire	*Outcomes*: - Direct and indirect comorbidity patterns from depression to 426 potential psychological, metabolic, and neurodegenerative disorders	The BDMM approach identified 320 direct comorbidity connections, and that model adequately differentiated direct relations from mediated relations among the diseases. The strongest comorbidities of depression were bipolar disorder, schizophrenia, and anxiety (the strongest) as psychological diseases, while diabetes and obesity were for metabolic disorders, and dementia, fibromyalgia, chronic fatigue, Parkinson, and migraines were for neurodegenerative disorders.

* Papers are reported in alphabetical order. SAT = Sensor-based artificial intelligence technology; CHR = clinical high-risk; ROD = recent onset depression; BDMM = Bayesian direct multimorbidity map.

## Data Availability

The datasets used and analyzed during the current study (the bibliography of included studies) are available from the corresponding author upon request.

## Supplementary Materials

The supporting information can be downloaded at https://www.mdpi.com/article/10.3390/bs15111483/s1. The Supplementary S1 contains our study’s full PRISMA checklist, according to Swartz’s recommendations (Swartz, 2021).

## Abbreviation

The following abbreviation is used in this manuscript:

AI	Artificial Intelligence
