# Supplementary material for: Can Artificial Intelligence Enhance European Emerging Adults’ Psychological Adjustment? A Scoping Review
Source: Behav Sci (Basel). 2025 Oct 31;15(11):1483. doi: 10.3390/bs15111483 (PMC12649690; doi:10.3390/bs15111483)
Supplement: Supplementary file 1 [file behavsci-15-01483-s001.zip › behavsci-3835571-supplementary.pdf]

Supplementary Materials

Search string

To conduct the literature research, we used the following three databases: Scopus, PsycINFO (via EBSCOhost), and PubMed.

The query string ran on the Scopus database was the following:

(TITLE-ABS-KEY ("psychological wellbeing" OR "mental health" OR "self-esteem" OR "emotional problems") AND ("university students" OR "college students" OR “university”) AND ("AI tools" OR "artificial intelligence" OR "machine learning" OR "chatbot" OR "digital intervention") AND ("Europe" OR "European countries" OR “Portugal” OR “Spain” OR “France” OR “Switzerland” OR “Austria” OR “Italy” OR “Germany” OR “Belgium” OR “Netherlands” OR “Croatia” OR “Greece” OR “Denmark” OR “Estonia” OR “Finland” OR “Sweden” OR “Hungary” OR “Ireland” OR “Latvia” OR “Lithuania” OR “Poland” OR “Slovakia” OR “Slovenia”) AND ("non-clinical sample" OR "healthy population" OR “community based community-based”) AND (PUBYEAR > 2014) AND (LIMIT-TO (LANGUAGE, "English")).

Additionally, we used the refine refined search tools provided by Scopus to limit the Year range from 2014 to 2025, the subject area (limited to Psychology, Neuroscience, Social Sciences, Health professions, and Arts and Humanities), the document type to articles and reviews, the country to European countries, the language to English, and the publication stage to final.

On PsycINFO, we ran the following query string:

((psychological AND wellbeing) OR (mental AND health) OR (self AND esteem) OR (emotional AND problems)) AND ((university AND students) OR (college AND students) OR university) AND (("ai" AND tools) OR (artificial AND intelligence AND tools) OR (machine AND learning) OR chatbot) AND (europe Europe OR european European OR eu OR (European AND countries)) AND ((non-clinical AND population) OR (healthy AND population) OR (community AND based)).

Additionally, we refined the research using the tools provided by EBSCO, to limit the Year range from 2014 to 2025, the document type to articles and reviews (excluding thesis), the country to European countries, the language to English, and the publication stage to final.

Lastly, on PubMed, we ran the following query string:

(("psychological wellbeing" OR "mental health" OR "self-esteem" OR "emotional problems") AND ("university students" OR "college students") AND ("AI tools" OR "artificial intelligence" OR "machine learning" OR "chatbot" OR "digital intervention") AND ("Europe" OR "European countries" OR “Portugal” OR “Spain” OR “France” OR “Switzerland” OR “Austria” OR “Italy” OR “Germany” OR “Belgium” OR “Netherlands” OR “Croatia” OR “Greece” OR “Denmark” OR “Estonia”

OR “Finland” OR “Sweden” OR “Hungary” OR “Ireland” OR “Latvia” OR “Lithuania” OR “Poland” OR “Slovakia” OR “Slovenia”) AND ("non-clinical sample" OR "healthy population" “community basedcommunity-based”) AND (2015:2025[dp]) AND (English[lang])).

A total of 139 records were found on Scopus, 11 on PsicINFO, and 17 on PubMed. To manage the identified records, we utilized the CADIMA online platform. After deleting duplicates (N=1), we identified 167 records. PRISMA 2020 Checklist Section and Topic Item # Checklist item Location where item is reported in Open Science Framework (https://osf.io/s9g5a - May 7th, 2025).

PRISMA 2020 Checklist

Section and Topic	Item #	Checklist item	Location where item is reported
**TITLE**			
Title	1	Identify the report as a systematic review.	p. 1
**ABSTRACT**			
Abstract	2	See the PRISMA 2020 for Abstracts checklist.	p.1
**INTRODUCTION**			
Rationale	3	Describe the rationale for the review in the context of existing knowledge.	pp. 1-3
Objectives	4	Provide an explicit statement of the objective(s) or question(s) the review addresses.	pp. 3-4
**METHODS**			
Eligibility criteria	5	Specify the inclusion and exclusion criteria for the review and how studies were grouped for the syntheses.	p. 4
Information sources	6	Specify all databases, registers, websites, organisations, reference lists and other sources searched or consulted to identify studies. Specify the date when each source was last searched or consulted.	p. 4
Search strategy	7	Present the full search strategies for all databases, registers and websites, including any filters and limits used.	p. 4
Selection process	8	Specify the methods used to decide whether a study met the inclusion criteria of the review, including how many reviewers screened each record and each report retrieved, whether they worked independently, and if applicable, details of automation tools used in the process.	pp. 4-5
Data collection process	9	Specify the methods used to collect data from reports, including how many reviewers collected data from each report, whether they worked independently, any processes for obtaining or confirming data from study investigators, and if applicable, details of automation tools used in the process.	pp. 5-6

Data items	10a	List and define all outcomes for which data were sought. Specify whether all results that were compatible with each outcome domain in each study were sought (e.g. for all measures, time points, analyses), and if not, the methods used to decide which results to collect.	p. 6
	10b	List and define all other variables for which data were sought (e.g. participant and intervention characteristics, funding sources). Describe any assumptions made about any missing or unclear information.	pp. 5-6
Study risk of bias assessment	11	Specify the methods used to assess risk of bias in the included studies, including details of the tool(s) used, how many reviewers assessed each study and whether they worked independently, and if applicable, details of automation tools used in the process.	None
Effect measures	12	Specify for each outcome the effect measure(s) (e.g. risk ratio, mean difference) used in the synthesis or presentation of results.	None
Synthesis methods	13a	Describe the processes used to decide which studies were eligible for each synthesis (e.g. tabulating the study intervention characteristics and comparing against the planned groups for each synthesis (item #5)).	p. 6
	13b	Describe any methods required to prepare the data for presentation or synthesis, such as handling of missing summary statistics, or data conversions.	None
	13c	Describe any methods used to tabulate or visually display results of individual studies and syntheses.	p. 6
	13d	Describe any methods used to synthesize results and provide a rationale for the choice(s). If meta-analysis was performed, describe the model(s), method(s) to identify the presence and extent of statistical heterogeneity, and software package(s) used.	None
	13e	Describe any methods used to explore possible causes of heterogeneity among study results (e.g. subgroup analysis, meta-regression).	None
	13f	Describe any sensitivity analyses conducted to assess robustness of the synthesized results.	None
Reporting bias assessment	14	Describe any methods used to assess risk of bias due to missing results in a synthesis (arising from reporting biases).	None
Certainty assessment	15	Describe any methods used to assess certainty (or confidence) in the body of evidence for an outcome.	None
**RESULTS**			
Study selection	16a	Describe the results of the search and selection process, from the number of records identified in the search to the number of studies included in the review, ideally using a flow diagram.	p. 6
	16b	Cite studies that might appear to meet the inclusion criteria, but which were excluded, and explain why they were excluded.	None
Study characteristics	17	Cite each included study and present its characteristics.	pp. 6-7
Risk of bias in studies	18	Present assessments of risk of bias for each included study.	None

Results of individual studies	19	For all outcomes, present, for each study: (a) summary statistics for each group (where appropriate) and (b) an effect estimate and its precision (e.g. confidence/credible interval), ideally using structured tables or plots.	pp. 6-13
Results of syntheses	20a	For each synthesis, briefly summarise the characteristics and risk of bias among contributing studies.	pp. 6-13
	20b	Present results of all statistical syntheses conducted. If meta-analysis was done, present for each the summary estimate and its precision (e.g. confidence/credible interval) and measures of statistical heterogeneity. If comparing groups, describe the direction of the effect.	pp. 6-13
	20c	Present results of all investigations of possible causes of heterogeneity among study results.	None
	20d	Present results of all sensitivity analyses conducted to assess the robustness of the synthesized results.	None
Reporting biases	21	Present assessments of risk of bias due to missing results (arising from reporting biases) for each synthesis assessed.	None
Certainty of evidence	22	Present assessments of certainty (or confidence) in the body of evidence for each outcome assessed.	None
**DISCUSSION**			
Discussion	23a	Provide a general interpretation of the results in the context of other evidence.	pp. 14-15
	23b	Discuss any limitations of the evidence included in the review.	pp. 14-15
	23c	Discuss any limitations of the review processes used.	pp. 14-15
	23d	Discuss implications of the results for practice, policy, and future research.	p. 15
**OTHER INFORMATION**			
Registration and protocol	24a	Provide registration information for the review, including register name and registration number, or state that the review was not registered.	p. 4
	24b	Indicate where the review protocol can be accessed, or state that a protocol was not prepared.	p. 16
	24c	Describe and explain any amendments to information provided at registration or in the protocol.	None
Support	25	Describe sources of financial or non-financial support for the review, and the role of the funders or sponsors in the review.	p. 16
Competing interests	26	Declare any competing interests of review authors.	p. 16
Availability of data, code and other materials	27	Report which of the following are publicly available and where they can be found: template data collection forms; data extracted from included studies; data used for all analyses; analytic code; any other materials used in the review.	pp. 4-5 & p. 16

Preferred Reporting Items for Systematic reviews and Meta-Analyses extension for Scoping Reviews (PRISMA-ScR) Checklist

SECTION	ITEM	PRISMA-ScR CHECKLIST ITEM	REPORTED ON PAGE #
**TITLE**			
Title	1	Identify the report as a scoping review.	p. 1
**ABSTRACT**			
Structured summary	2	Provide a structured summary that includes (as applicable): background, objectives, eligibility criteria, sources of evidence, charting methods, results, and conclusions that relate to the review questions and objectives.	p. 1
**INTRODUCTION**			
Rationale	3	Describe the rationale for the review in the context of what is already known. Explain why the review questions/objectives lend themselves to a scoping review approach.	pp. 1-4
Objectives	4	Provide an explicit statement of the questions and objectives being addressed with reference to their key elements (e.g., population or participants, concepts, and context) or other relevant key elements used to conceptualize the review questions and/or objectives.	pp. 4-5
**METHODS**			
Protocol and registration	5	Indicate whether a review protocol exists; state if and where it can be accessed (e.g., a Web address); and if available, provide registration information, including the registration number.	p.5
Eligibility criteria	6	Specify characteristics of the sources of evidence used as eligibility criteria (e.g., years considered, language, and publication status), and provide a rationale.	p.5-6
Information sources	7	Describe all information sources in the search (e.g., databases with dates of coverage and contact with authors to identify additional sources), as well as the date the most recent search was executed.	p.6-7
Search	8	Present the full electronic search strategy for at least 1 database, including any limits used, such that it could be repeated.	pp. 1-2 of Supplementary material
Selection of sources of evidence	9	State the process for selecting sources of evidence (i.e., screening and eligibility) included in the scoping review.	p.7
Data charting process	10	Describe the methods of charting data from the included sources of evidence (e.g., calibrated forms or forms that have been tested by the team before their use, and whether data charting was done independently or in duplicate) and any processes for obtaining and confirming data from investigators.	p.7
Data items	11	List and define all variables for which data were sought and any assumptions and simplifications made.	p.7
Critical appraisal of individual sources of evidence	12	If done, provide a rationale for conducting a critical appraisal of included sources of evidence; describe the methods used and how this information was used in any data synthesis (if appropriate).	None
Synthesis of results	13	Describe the methods of handling and summarizing the data that were charted.	p.7

SECTION	ITEM	PRISMA-ScR CHECKLIST ITEM	REPORTED ON PAGE #
**RESULTS**			
Selection of sources of evidence	14	Give numbers of sources of evidence screened, assessed for eligibility, and included in the review, with reasons for exclusions at each stage, ideally using a flow diagram.	p.7
Characteristics of sources of evidence	15	For each source of evidence, present characteristics for which data were charted and provide the citations.	p.8
Critical appraisal within sources of evidence	16	If done, present data on critical appraisal of included sources of evidence (see item 12).	None
Results of individual sources of evidence	17	For each included source of evidence, present the relevant data that were charted that relate to the review questions and objectives.	pp.8-10
Synthesis of results	18	Summarize and/or present the charting results as they relate to the review questions and objectives.	pp.11-12
**DISCUSSION**			
Summary of evidence	19	Summarize the main results (including an overview of concepts, themes, and types of evidence available), link to the review questions and objectives, and consider the relevance to key groups.	pp.13-14
Limitations	20	Discuss the limitations of the scoping review process.	p.14
Conclusions	21	Provide a general interpretation of the results with respect to the review questions and objectives, as well as potential implications and/or next steps.	p.14
**FUNDING**			
Funding	22	Describe sources of funding for the included sources of evidence, as well as sources of funding for the scoping review. Describe the role of the funders of the scoping review.	p.14
